# Prescription Department

**Published:** 1892-04

**Authors:** 


NERVOUS DYSPEPSIA.—

R. Strychninæ sulph., gr. 1-36.
Acidi phosphorici, dil. mxx.
Glycerini, dr. ss.
Aquæ, q. s., dr. j.

S. At one dose, three times a day, before meals.—*Times and Register.*

CONVULSIONS OF TEETHING.—

R. Chloral hydrat., gr. xv.
Potass. bromide, dr. j.
Syrup simplic, f dr. v.
Aq. distillat., f oz. ij.

M. Sig. Teaspoonful every three hours.—*Kinder-Arzt.—St. Louis Clinique.*

SWOLLEN EYES.—If the eyes are swollen use on them:

R. Acid boracic, gr. xij.
Aquæ camphoræ,
Aquæ dest., aa oz. ii.

Bathe and drop in the eyes frequently.—*Med. Brief.*

LAXATIVE FOR CHILDREN.—

R. Manna, dr. vi.
Magnesia,
Sulphur, aa oz. iss.
Mel., dr. vi.

M. Sig. One or two dessertspoonfuls in a cup of warm milk or weak tea —FERRAND in *Gaz. de Gyn.*

TO ABORT BOILS.—

R. Camphoræ, 2 drachms.

Chloroformi, ½ ounce.

M. et solve. Sig. Apply with tip of finger hourly for one day. It will abort all boils when used before suppuration has begun.

FOR COUGH.—For severe coughs and colds, when the ordinary expectorants fail to accomplish their object, the following has often been found serviceable:

R. Ext. yerb santa fl.
Ext. grindelia robusta aa oz. ss.
Syr. prunus virg., oz. ij.

M. Sig. Teaspoonful every two or three hours.—*Med. Summary.*

In *neuralgic cephalalgia* and in the *early stage of la grippe*, when the patient complains of pains and aches from head to foot, the following has answered admirably. It also causes a reduction of temperature in la grippe:

R. Quininæ sulph., gr. ix.
Antipyrine, gr. xviij.
Ext. hyoscyami, gr. iij.

M. Ft. capsul. No. vj. Sig. One capsule every two or three hours.—*Ex.*

For Cough.—

R. Potassii iodidi, 1 drachm.
Potassii chloras, 1 drachm.
Liq. acidi carb., 4 minims.
Syr. aurantii, 2 ounces.

M. Sig. Teaspoonful every four hours until relief is obtained.

I find the above prescription to be the best cough mixture I ever tried. It will stop a cough of long standing in three days.—Watt, in *Med. Brief.*

Incontinence of Urine.—For *incontinence of urine* in children due to exposure to cold, Professor Hare recommends the following treatment:

Where the urine is high-colored and concentrated, and the child has fever, give—

R. Tinct aconit., gtt. xij to xxiv.
Spt. ætheris nitrosi, f. dr. ij to iv
Liq. potass. citratis, ad f oz. vj.
M. Sig. Drachms ii every three hours.

After the urine has become more diluted, belladonna can be given with advantage, to allay the irritation and spasm of the bladder.

Or when the incontinence is due to paralysis of the bladder, give—

R. Extract nucis vomicæ, gr. ij.
Acid arseniosi, gr. 1-3.
M Fiant pil. xx.
Sig. One pill three times a day.—*Coll and Clin. Record.*

Pruritis Ani. Doctor Joseph M. Matthews has obtained excellent results from—

R. Benz. oxide zinc oint.
Campho-phenique, aa oz. ss.
M. Apply as often as necessary.

The campho-phenique may also be used pure, without detriment to skin or mucous membrane.—*Med. Bulletin*, February, '92.

